# Sagittal, Vertical, and Transverse Skeletal Characteristics in Individuals with Impacted Maxillary Canines: A Retrospective Study

**DOI:** 10.3390/jcm15041466

**Published:** 2026-02-13

**Authors:** Nuri Can Tanrısever, Mehmet Okan Akçam

**Affiliations:** Department of Orthodontics, School of Dentistry, University of Ankara, 06560 Ankara, Türkiye

**Keywords:** cephalometry, cuspid, malocclusion, tooth, impacted, orthodontics

## Abstract

**Background/Objectives:** Maxillary canine impaction is a multifactorial condition that may adversely affect esthetics, function, and occlusal stability. Although various etiologic factors have been proposed, the skeletal characteristics observed in individuals with maxillary canine impaction remain incompletely understood. This study aimed to evaluate sagittal, vertical, and transverse skeletal characteristics in individuals with impacted maxillary canines using lateral and posteroanterior cephalometric radiographs. **Methods:** This retrospective study included lateral and posteroanterior cephalometric radiographs of 100 individuals (mean age: 15.85 ± 1.69 years) with at least one impacted maxillary canine. Sagittal skeletal relationships were assessed using SNA, ANB, and Wits appraisal, while vertical relationships were evaluated using the GoGn/SN and FH/MP angles. Transverse skeletal dimensions (JR–JL, JR–ZAg, JL–ZAg, and Ag–Ag) were evaluated and compared with a matched control group without impacted maxillary canines using independent-samples *t*-tests. **Results:** Sagittal and vertical evaluations demonstrated distributional patterns of skeletal classifications within the impaction sample. Transverse analysis revealed significantly reduced maxillary width (JR–JL) and increased maxillomandibular transverse measurements (JR–ZAg and JL–ZAg) in the impaction group compared with the control group (*p* < 0.001). **Conclusions:** Sagittal and vertical skeletal findings represent distributional characteristics among affected individuals, whereas statistically significant differences were identified only for transverse skeletal dimensions. These findings underscore the clinical relevance of transverse skeletal assessment in individuals with impacted maxillary canines. Prospective studies are required before such observations can be translated into risk prediction.

## 1. Introduction

Tooth eruption is a complex and multifactorial biological process regulated by the coordinated interaction of genetic, local, and systemic factors. Disruptions in this process may result in delayed eruption or complete failure of eruption, ultimately leading to tooth impaction. Abron et al. defined impaction as a deceleration in the normal eruption rate of a tooth, whereas Lindauer et al. described an impacted tooth as one that fails to erupt after the completion of root development or when the contralateral homologous tooth has erupted at least six months earlier [1,2]. These definitions suggest that tooth impaction should be regarded not as a single event, but as the final outcome of a disturbed eruption pathway.

The etiology of tooth impaction is multifactorial and may involve the combined or independent effects of local, systemic, and genetic factors acting at different stages of the eruption mechanism [3]. Local factors include space deficiency, abnormal eruption pathways, and mechanical interference from adjacent teeth, with insufficient arch space—most commonly resulting from an arch–tooth size discrepancy—being identified as a primary local factor associated with maxillary canine impaction. Among permanent teeth, third molars are the most frequently impacted, followed by permanent canines [4]. The prevalence of impacted canines has been reported to range between 1% and 4%, with maxillary canines being affected more frequently than mandibular canines [5,6,7,8]. These epidemiological estimates and classical concepts continue to be supported and discussed in contemporary systematic reviews and large clinical studies evaluating maxillary canine impaction [9].

In the context of maxillary canine impaction, two principal etiological concepts have been proposed: the guidance theory and genetic predisposition. The guidance theory suggests that disturbances in local eruptive guidance—such as anomalies of the lateral incisor or insufficient arch space—may redirect the canine eruption pathway, whereas the genetic theory emphasizes inherited developmental patterns affecting tooth position and craniofacial morphology. Current evidence indicates that these mechanisms are not mutually exclusive and may interact with underlying skeletal characteristics, contributing to the multifactorial nature of maxillary canine impaction [3].

Permanent maxillary canines play a crucial role in dental esthetics, functional occlusion, and guidance during mandibular movements due to their long root morphology, robust periodontal support, and strategic position within the dental arch [10]. Impaction of these teeth may lead to significant clinical consequences, including root resorption of adjacent teeth, periodontal deterioration, and disturbances in occlusal stability [11]. As a result, impacted maxillary canines often require complex orthodontic and/or surgical interventions, underscoring the importance of early identification of predisposing factors and skeletal characteristics associated with this condition [12].

From a diagnostic perspective, maxillary canine impaction is routinely assessed through clinical examination in combination with panoramic and cephalometric radiography. In selected complex cases, cone-beam computed tomography (CBCT) may provide additional three-dimensional information regarding tooth position and adjacent anatomical structures [13]. However, most previous investigations have primarily focused on tooth localization, dentoalveolar characteristics, or treatment difficulty rather than on comprehensive evaluation of the underlying skeletal relationships [14,15,16,17]. Moreover, the routine use of CBCT for screening purposes remains limited due to radiation exposure and accessibility considerations.

Previous studies have suggested that skeletal patterns in the sagittal, vertical, and transverse dimensions may be associated with variations in the eruption pathway of maxillary canines. In particular, transverse skeletal deficiency, reflected by reduced maxillary basal or alveolar width, has been relatively consistently observed in individuals with impacted maxillary canines [18]. In contrast, the influence of sagittal and vertical skeletal relationships remains controversial, with inconsistent and sometimes conflicting findings reported in the literature [19,20]. Given that maxillary canine eruption occurs within an integrated sagittal, vertical, and transverse skeletal framework, a comprehensive evaluation of these dimensions is important for improving etiological understanding and supporting clinical assessment.

Therefore, the aim of this retrospective study was to evaluate the association and distribution of sagittal, vertical, and transverse skeletal patterns in individuals with impacted maxillary canines using lateral and posteroanterior cephalometric radiographs. Rather than establishing causal or predictive risk relationships for all skeletal dimensions, this study sought to describe skeletal characteristics observed in affected individuals and to identify skeletal dimensions that demonstrate more consistent associations with maxillary canine impaction.

## 2. Materials and Methods

### 2.1. Study Design and Sample

This single-center retrospective study was conducted using archived lateral and posteroanterior cephalometric radiographs obtained from the Department of Orthodontics, Faculty of Dentistry, Ankara University. Patient records collected between June 2023 and December 2024 were screened. A consecutive sampling approach was used, whereby all available records within this period that met the inclusion criteria were considered eligible for analysis.

The study included cephalometric radiographs of 100 individuals (mean age: 15.85 ± 1.69 years; 47 males, 53 females) presenting with at least one impacted maxillary canine. Records were excluded if radiographs were incomplete or of insufficient quality for reliable landmark identification, if patients had a history of previous orthodontic treatment, craniofacial syndromes, cleft lip and palate, or systemic conditions affecting dental development. Cases with missing key cephalometric measurements were excluded from the analysis; no data imputation was performed.

All radiographs were acquired by the same radiographic technician using the same digital radiographic system (Carestream Dental 8100, Carestream Health Inc., Rochester, NY, USA). Images were obtained with patients positioned in a standardized neutral head position in accordance with the manufacturer’s instructions.

### 2.2. Diagnostic Criteria for Maxillary Canine Impaction

**Figure 1:**
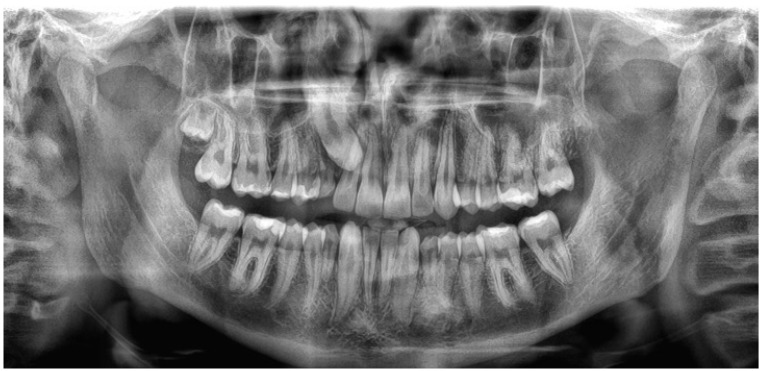
Representative panoramic radiograph illustrating the diagnosis of impacted maxillary canine based on eruption status and root development.

The diagnosis of maxillary canine impaction was established using a combination of chronological eruption age, intraoral photographs, and panoramic radiographs (Figure 1). A maxillary canine was considered impacted if it met all of the following criteria:

(1) Absence from the dental arch at least six months beyond the expected eruption age;
(2) Absence on intraoral photographs;
(3) Failure to erupt despite completion of at least three-quarters of root development as observed on panoramic radiographs.

The criterion of eruption delay was applied as a screening threshold rather than an absolute developmental marker, acknowledging inter-individual and population-related variation in eruption timing. Radiographic assessment of root development was used to support the diagnosis, and cases were included at a mean chronological age of 15.85 ± 1.69 years, a developmental stage at which maxillary canine eruption is expected to have occurred in the studied population. Accordingly, the combined use of eruption status and radiographic maturation was intended to reduce potential misclassification between delayed eruption and definitive impaction under retrospective conditions.

These criteria were selected to reflect routine clinical screening conditions rather than to provide a detailed three-dimensional characterization of impaction morphology. In the present study, the diagnostic framework was applied to identify the presence of maxillary canine impaction at a global level.

Detailed characterization of impaction subtypes, including buccal or palatal position, unilateral or bilateral involvement, severity, or proximity to adjacent tooth roots, was not performed in a standardized manner and therefore was not included in the analysis. Accordingly, skeletal assessments were conducted without stratification according to specific impaction subtypes.

### 2.3. Sagittal and Vertical Skeletal Assessment

**Figure 2:**
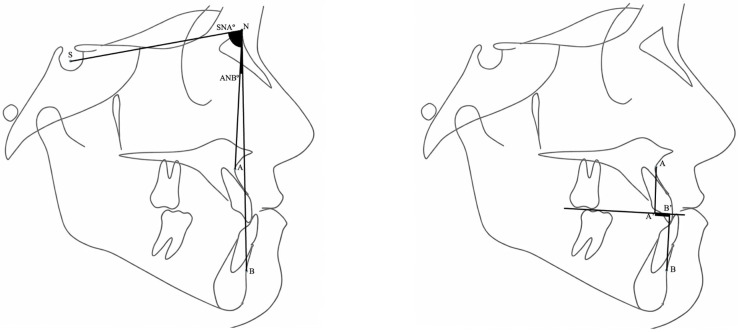
Sagittal skeletal assessment on lateral cephalometric radiographs using SNA°, ANB°, and Wits appraisal. Wits appraisal represents the linear distance between points A and B projected onto the functional occlusal plane.

Sagittal skeletal relationships were evaluated on lateral cephalometric radiographs using SNA°, ANB°, and Wits appraisal according to Steiner analysis and were classified as skeletal Class I, Class II, or Class III (Figure 2). Skeletal classification was defined as Class I (ANB = 0–4°), Class II (ANB > 4°), and Class III (ANB < 0°).

**Figure 3:**
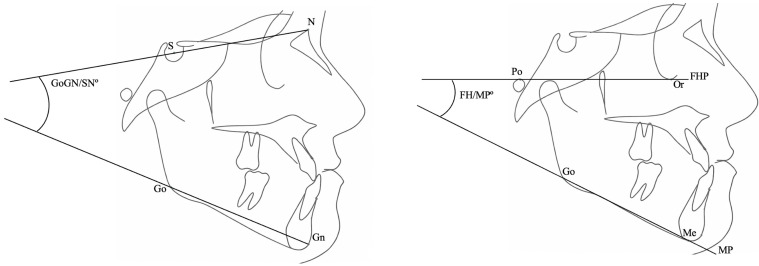
Vertical skeletal assessment using GoGn/SN° and FH–MP° angles on lateral cephalometric radiographs. The Frankfort horizontal plane (FHP) was defined by the Po–Or line, and the mandibular plane (MP) was defined by the Go–Me line.

Vertical skeletal patterns were assessed using the GoGn/SN° and FH–MP° (PoOr-GoMe) angles and categorized as hypodivergent, normodivergent, or hyperdivergent (Figure 3). Vertical classification was defined as hypodivergent (GoGn/SN < 26°), normodivergent (26–38°), and hyperdivergent (>38°), with the FH–MP angle used as a complementary parameter.

### 2.4. Transverse Skeletal Assessment

For the evaluation of transverse skeletal relationships, measurements were compared with a control group. The control group consisted of 100 individuals without impacted maxillary canines, selected from the same institutional archive and matched to the study group by age and sex using frequency matching (±1 year). Controls with posterior crossbite were excluded to minimize the influence of dentoalveolar transverse compensations and functional mandibular shifts on skeletal transverse measurements. All control radiographs were acquired using the same imaging device and protocol as the study group.

Other dental characteristics such as crowding severity or tooth size–arch length relationships were not used as exclusion criteria for the control group due to non-standardized documentation in archived records.

**Figure 4:**
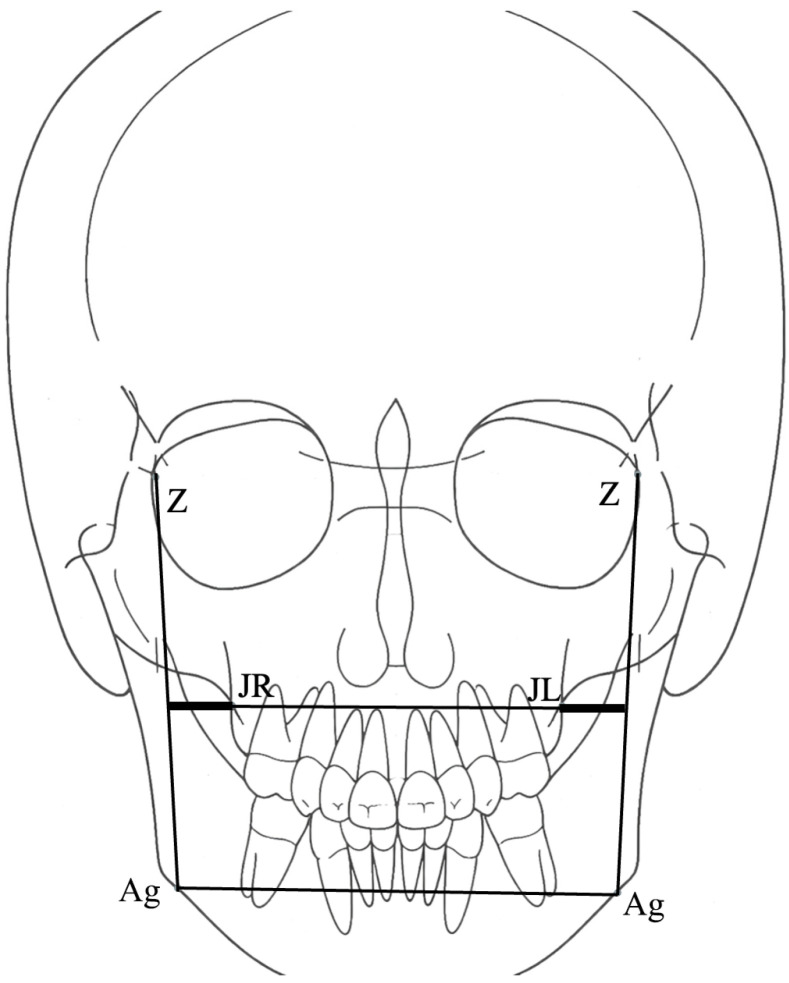
Transverse skeletal measurements on posteroanterior cephalometric radiographs: maxillary width (JR–JL), maxillomandibular widths (JR–ZAg and JL–ZAg), and mandibular width (Ag–Ag).

Transverse skeletal relationships were evaluated on posteroanterior cephalometric radiographs using standard transverse measurements, including maxillary width (JR–JL), maxillomandibular widths (JR–ZAg and JL–ZAg), and mandibular width (Ag–Ag) (Figure 4). In this analysis, the Z point was anatomically defined as the intersection of the zygomaticofrontal suture with the orbital rim, ensuring consistent landmark identification for transverse skeletal assessment. JR and JL were defined as the right and left jugular points representing the lateral maxillary skeletal boundaries, while Ag denoted the antegonial point used to assess mandibular transverse width.

### 2.5. Cephalometric Measurements and Reliability

To ensure measurement accuracy, the 10 mm reference distance of the cephalostat calibration rod on archived radiographs was scaled to 1 cm within the software. All cephalometric landmarks and measurements were identified according to standard anatomical definitions used in conventional cephalometric analyses, including Steiner analysis and the Wits appraisal. All angular and linear measurements were performed by a single examiner (N.C.T.) using Dolphin Imaging software (Version 11.95.08.50 Premium, Dolphin Imaging & Management Solutions, Chatsworth, CA, USA) and AutoCAD software (AutoCAD 2025, Autodesk Inc., San Francisco, CA, USA).

Intra-observer reliability was assessed by re-measuring 20 randomly selected lateral and posteroanterior cephalometric radiographs after a two-week interval. Intraclass correlation coefficients (ICC) were calculated for all measurements, with ICC values ranging from 0.91 to 0.97, indicating excellent intra-observer reliability.

Inter-observer reliability assessment was not performed because all cephalometric measurements were intentionally conducted by a single experienced examiner to minimize variability related to landmark identification and measurement technique. Given the retrospective design and the use of archived radiographs, inclusion of a second independent examiner was not feasible within the standardized measurement protocol. However, all measurements were performed according to predefined landmark definitions and standardized measurement protocols to reduce potential measurement bias.

### 2.6. Ethical Approval

This retrospective study was approved by the Ethics Committee of Ankara University Faculty of Dentistry (Meeting No: 9, Approval Date: 5 May 2025). Due to the retrospective design and use of archived data, informed consent was waived by the ethics committee. All procedures were conducted in accordance with the Declaration of Helsinki and relevant ethical guidelines.

### 2.7. Statistical Analysis

Statistical analyses were performed using IBM SPSS Statistics software (Version 26; IBM Corp., Armonk, NY, USA). Data normality was assessed using the Shapiro–Wilk test. Variables demonstrating normal distribution were analyzed using parametric statistical tests, whereas non-parametric alternatives were applied when normality assumptions were not met.

Within the impaction sample, groups were formed based on established cutoff values for SNA°, ANB°, GoGn/SN°, and FH–MP° measurements. Differences in continuous skeletal measurements among these categorical groups were evaluated using one-way analysis of variance (ANOVA). When the assumptions for ANOVA were not satisfied, the Kruskal–Wallis test was used as a non-parametric alternative. Frequency and percentage distributions were calculated for categorical skeletal classifications. Because Wits appraisal cutoff values differ by sex, analyses based on Wits measurements were performed separately for males and females. Analyses related to sagittal and vertical skeletal dimensions were therefore descriptive in nature, intended to characterize distributional patterns rather than to test inferential associations.

Transverse skeletal measurements were compared between the study and control groups using independent-samples *t*-tests. In addition to *p* values, effect sizes were calculated using Cohen’s *d* based on pooled standard deviations to quantify the magnitude of between-group differences. Effect sizes were interpreted according to Cohen’s criteria as small (0.2), moderate (0.5), or large (0.8).

Given the exploratory aim of the transverse comparisons and the limited number of predefined transverse outcomes (JR–JL, JR–ZAg, JL–ZAg, and Ag–Ag), no formal adjustment for multiple comparisons was applied, and the results should be interpreted with consideration of potential type I error inflation.

All statistical analyses were conducted at a 95% confidence level, and a *p*-value < 0.05 was considered statistically significant. No multivariable modeling approach (e.g., regression analysis) was employed to adjust for potential confounding factors beyond age and sex matching. Accordingly, statistically significant findings—particularly for transverse skeletal measurements—represent unadjusted group-level differences.

An a priori sample size calculation was performed for planned comparisons of continuous skeletal measurements across categorical variables with more than two groups within the impaction sample (one-way ANOVA framework). Assuming a large effect size (Cohen’s f = 0.60), a significance level of α = 0.05, and a desired statistical power of 90% (1–β = 0.90), the minimum required sample size was calculated as n = 39. The final impaction sample size (n = 100) exceeded this requirement for the intended multi-group analyses.

## 3. Results

A total of 100 individuals with at least one impacted maxillary canine were included in the analysis. Descriptive statistics of sagittal and vertical skeletal measurements are presented in Table 1.

### 3.1. Sagittal Skeletal Distribution

Sagittal skeletal relationships were evaluated using SNA°, SNB°, and ANB° angles and Wits appraisal. Based on SNA°, the sagittal position of the maxilla within the impaction sample was distributed as follows: 59% of individuals demonstrated a normally positioned maxilla, while retrusive and protrusive maxillary positions accounted for 21% and 20% of cases, respectively. According to ANB°, skeletal Class I pattern constituted the largest proportion of the impaction sample (56%), followed by Class III (25%) and Class II (19%) patterns (Table 2).

When assessed using Wits appraisal, sagittal skeletal classifications showed sex-related variation within the impaction sample. In males, skeletal Class I pattern represented the largest proportion (46.8%), whereas Class II pattern accounted for the smallest proportion (23.4%). In females, skeletal Class I pattern accounted for the largest proportion of the impaction sample (41.5%), whereas skeletal Class III pattern constituted the smallest proportion (22.7%) (Table 3).

### 3.2. Vertical Skeletal Distribution

Vertical skeletal patterns were evaluated using GoGn/SN° and FH–MP° angles. Based on GoGn/SN°, the distribution of vertical skeletal patterns within the impaction sample showed that hyperdivergent patterns accounted for 59% of cases, followed by normodivergent (29%) and hypodivergent (12%) patterns. Assessment using the FH–MP° angle demonstrated a comparable distribution, with hyperdivergent patterns accounting for 51% of cases (Table 4).

### 3.3. Transverse Skeletal Comparison

In contrast to sagittal and vertical assessments, comparative statistical analysis was performed for transverse skeletal measurements. Independent-samples *t*-tests demonstrated statistically significant differences between the impaction and control groups for maxillary width (JR–JL) and maxillomandibular transverse measurements (JR–ZAg and JL–ZAg) (*p* < 0.001).

Specifically, the impaction group exhibited a reduced maxillary width (JR–JL) compared with the control group, with a moderate effect size (Cohen’s d = −0.68). In contrast, maxillomandibular transverse measurements (JR–ZAg and JL–ZAg) were greater in the impaction group, demonstrating a large effect size for JR–ZAg (Cohen’s d = 1.15) and a moderate effect size for JL–ZAg (Cohen’s d = 0.56) (Table 5).

No statistically significant difference was observed between the groups for mandibular width (Ag–Ag) (*p* = 0.824), and the corresponding effect size was negligible (Cohen’s d = 0.03) (Table 5).

## 4. Discussion

The present study aimed to evaluate sagittal, vertical, and transverse skeletal characteristics in individuals with impacted maxillary canines using lateral and posteroanterior cephalometric radiographs. The findings demonstrate that sagittal and vertical skeletal dimensions exhibit distinct distributional patterns within the impaction sample, whereas a statistically significant difference was identified only for transverse skeletal dimensions when compared with a control group.

In the sagittal dimension, the skeletal Class I pattern constituted the largest proportion of individuals within the impaction sample, followed by Class III and Class II patterns. Similar distributional tendencies were observed using Wits appraisal, with minor sex-related variations. These observations differ from previous reports describing a higher prevalence of maxillary canine impaction in skeletal Class III individuals, a condition often associated with transverse maxillary deficiency [12,21], while other investigations have reported no meaningful differences between skeletal Class I and Class III patterns [22]. Importantly, the present findings reflect distributional characteristics among affected individuals rather than comparative prevalence across skeletal classes, as no non-impaction comparator or population-based denominator was available.

The discrepancies reported in the sagittal literature may be attributable to variations in sample size, skeletal classification criteria, and measurement methodologies, as well as the frequent coexistence of transverse maxillary insufficiency within certain sagittal skeletal patterns. Accordingly, sagittal skeletal assessment alone appears insufficient to explain the multifactorial nature of maxillary canine impaction. From a clinical standpoint, this distribution highlights that maxillary canine impaction may also be encountered in patients with sagittally balanced skeletal relationships, underscoring the importance of maintaining clinical vigilance during routine orthodontic assessment regardless of sagittal skeletal class [23].

With respect to the vertical dimension, hyperdivergent growth patterns accounted for a larger proportion of the impaction sample compared with normodivergent and hypodivergent patterns. These distributional findings are consistent with previous studies reporting increased vertical skeletal measurements, including GoGn/SN°, Y-axis, and gonial angle, in individuals with impacted maxillary canines [24,25]. Conversely, other authors have described a greater occurrence of canine impaction in hypodivergent individuals or in those presenting with deep bite patterns [26,27,28,29]. Such inconsistencies may reflect differences in age distribution, vertical classification thresholds, and sample composition across studies. As with sagittal findings, the present vertical results should be interpreted as descriptive characteristics rather than indicators of skeletal predisposition.

In contrast to sagittal and vertical assessments, transverse skeletal evaluation revealed a statistically significant difference between individuals with impacted maxillary canines and controls. Specifically, reduced maxillary width and altered maxillomandibular transverse relationships were observed in the impaction group. Notably, effect size analysis indicated that the magnitude of these differences was moderate for maxillary width and ranged from moderate to large for maxillomandibular transverse measurements, supporting the clinical relevance of the observed transverse skeletal discrepancies. These findings support the concept that transverse skeletal deficiency may influence the eruption pathway of maxillary canines by limiting basal and alveolar bone width and contributing to a narrow palatal morphology. Comparable associations between reduced maxillary width and maxillary canine impaction have been reported using both two-dimensional and three-dimensional imaging modalities [14,30,31]. From a clinical perspective, recognition of transverse skeletal deficiency on routine posteroanterior cephalometric radiographs may prompt closer surveillance of canine eruption and earlier consideration of interceptive orthodontic approaches, such as assessment for maxillary expansion, within the context of comprehensive orthodontic evaluation rather than as a standalone decision criterion.

Nevertheless, the relationship between transverse maxillary dimensions and canine impaction is not uniformly supported in the literature. Several studies have reported no significant transverse differences between individuals with and without impacted maxillary canines, while others have described greater maxillary width in the impaction group [32,33,34,35,36]. These conflicting results may be related to heterogeneity in imaging techniques, landmark definitions, age ranges, and inclusion criteria, as well as differences in impaction characteristics such as unilateral versus bilateral involvement and buccal versus palatal localization. Moreover, sex-related biological factors, including sexual dimorphism and differences in craniofacial growth timing, may act as potential modifiers of transverse skeletal development and could contribute to variability across study populations.

In addition, unmeasured dental factors such as arch length discrepancy, incisor anomalies, and tooth size–arch length relationships may influence transverse skeletal dimensions and act as residual confounders, potentially contributing to the inconsistent findings reported across studies.

Taken together, the present findings suggest that transverse skeletal deficiency represents the most consistent skeletal characteristic distinguishing individuals with impacted maxillary canines from unaffected controls, whereas sagittal and vertical skeletal dimensions exhibit more variable distributional patterns within the impaction sample. The combined use of routine lateral and posteroanterior cephalometric radiographs allows comprehensive assessment of skeletal relationships across three planes within a standardized diagnostic framework.

This study has several limitations that should be acknowledged. Its retrospective and single-center design may limit generalizability. Moreover, sagittal and vertical skeletal dimensions were evaluated using distribution-based analyses without comparative statistical testing, precluding inferential conclusions regarding skeletal susceptibility. Unlike sagittal and vertical dimensions, which can be interpreted using established normative reference values from conventional cephalometric analyses, transverse skeletal measurements lack universally accepted reference standards, thereby necessitating comparison with a matched control group. Because no formal adjustment for multiple comparisons was applied, the statistically significant transverse findings should be interpreted as exploratory and hypothesis-generating rather than confirmatory. However, the inclusion of effect size estimates allows a more nuanced interpretation of the findings beyond statistical significance alone. Importantly, potential effect modifiers—including unilateral or bilateral impaction, buccal or palatal position of the impacted canine, and dental crowding or arch length discrepancy—were not analyzed. These factors are known to influence both skeletal morphology and impaction patterns and may obscure or modify the underlying skeletal signal, thereby limiting internal validity.

Furthermore, impaction morphology itself may differentially interact with skeletal dimensions. Palatally impacted canines have been more frequently associated with transverse maxillary deficiency, whereas buccally impacted canines may reflect localized dentoalveolar constraints rather than basal skeletal discrepancies. Similarly, bilateral impaction may indicate a stronger skeletal component compared with unilateral involvement, which may be more influenced by local eruptive factors.

In addition, inter-observer reliability was not assessed, which may limit the generalizability of measurement reproducibility across different examiners. Nevertheless, the use of a single calibrated examiner and standardized landmark definitions likely contributed to consistent measurements across the dataset, partially mitigating this limitation.

Taken together, future prospective and multicenter studies incorporating standardized subtype classification, comprehensive assessment of potential effect modifiers, and multivariable analytical approaches are warranted.

## 5. Conclusions

This study demonstrates that sagittal and vertical skeletal dimensions show distinct distributional patterns among individuals with impacted maxillary canines, whereas a statistically significant difference was identified only for transverse skeletal dimensions when compared with unaffected controls. In this respect, transverse skeletal deficiency appears to represent the most consistent skeletal characteristic distinguishing individuals with impacted maxillary canines. However, transverse skeletal deficiency should be interpreted as a contributory feature within a multifactorial diagnostic framework rather than as an independent or standalone predictor of maxillary canine impaction.

Findings related to sagittal and vertical dimensions should be interpreted as descriptive rather than inferential. While routine lateral and posteroanterior cephalometric radiographs enable comprehensive assessment of skeletal relationships, prospective studies with adequate control of confounding factors are required before such findings can be translated into early risk prediction.

## Figures and Tables

**Table 1:** Descriptive Statistics of Sagittal and Vertical Skeletal Measurements.

Measurements	Min-Max	Mean ± SD
SNA°	73–89	81.21 ± 3.73
SNB°	68–89	79.21 ± 5.27
ANB°	−5–8	2.02 ± 3.41
Wits (mm)	−9–8	0.27 ± 3.57
GoGN/SN°	14.6–44.3	32.42 ± 5.67
FH/MP°	7.7–34.9	24.9 ± 5.48

Abbreviations: SD, standard deviation.

**Table 2:** Distribution of Impacted Maxillary Canines According to Skeletal Sagittal Classification within the Impaction Sample.

Parameters	Classification (°)	n (%)
SNA°	80–84° (normal)	59 (59%)
	>84° (protrusive maxilla)	20 (20%)
	<80° (retrusive maxilla)	21 (21%)
ANB°	0–4° (Class I)	56 (56%)
	>4° (Class II)	19 (19%)
	<0° (Class III)	25 (25%)

Abbreviations: n, number of subjects.

**Table 3:** Distribution of Skeletal Sagittal Classifications Based on Wits Appraisal within the Impaction Sample, Stratified by Sex.

Gender (n)	Wits Appraisal (mm)	n (%)
Male (n = 47)	−3 to −1 (Class I)	22 (46.8%)
	>−1 (Class II)	11 (23.4%)
	<−3 (Class III)	14 (29.8%)
Female (n = 53)	−2 to 0 (Class I)	22 (41.5%)
	>0 (Class II)	19 (35.8%)
	<−2 (Class III)	12 (22.7%)

**Table 4:** Distribution of Skeletal Vertical Classifications within the Impaction Sample.

Parameters	Classification (°)	n (%)
GoGN/SN°	<26 (hypodivergent)	12 (12%)
	26–38 (normodivergent)	29 (29%)
	>38 (hyperdivergent)	59 (59%)
FH/MP°	<18 (hypodivergent)	16 (16%)
	18–26 (normodivergent)	33 (33%)
	>26 (hyperdivergent)	51 (51%)

**Table 5:** Comparison of Maxillary and Mandibular Transversal Measurements in the Study and Control Groups.

Measurements (mm)	Study Group (n = 100) Mean ± SD	Control Group (n = 100) Mean ± SD	*p*	Cohen’s d
JR–JL	57.96 ± 3.36	61.44 ± 6.42	<0.001 *	−0.68
JR–ZAg	12.72 ± 1.98	10.87 ± 1.12	<0.001 *	1.15
JL–ZAg	12.22 ± 1.72	11.32 ± 1.48	<0.001 *	0.56
AG–AG	78.13 ± 5.59	77.94 ± 6.61	0.824	0.03

Significance level: * *p* ˂ 0.05. Independent samples *t*-test was used. Cohen’s d was calculated using pooled standard deviation to assess effect size between groups. Effect size interpretation followed Cohen’s criteria: 0.2 = small, 0.5 = moderate, 0.8 = large.

## Data Availability

The data presented in this study are not publicly available due to ethical and privacy restrictions, as they are derived from anonymized patient radiographic records obtained from institutional archives. Data may be made available from the corresponding author upon reasonable request and with permission of the relevant ethics committee.

## Abbreviations

ANB	A point–Nasion–B point angle
Ag–Ag	Mandibular width
CBCT	Cone-beam computed tomography
FH–MP	Frankfort horizontal plane–mandibular plane angle
GoGn/SN	Gonion–Gnathion to Sella–Nasion angle
ICC	Intraclass correlation coefficient
JR–JL	Maxillary width
JR–ZAg	Right maxillomandibular width
JL–ZAg	Left maxillomandibular width
SD	Standard deviation
SNA	Sella–Nasion–A point angle
SNB	Sella–Nasion–B point angle
SPSS	Statistical Package for the Social Sciences
Wits	Wits appraisal
